# Editorial: Exploring the role of stem cells in bone health and regeneration

**DOI:** 10.3389/fmed.2026.1809426

**Published:** 2026-03-09

**Authors:** Elena Stocco, Adam Qingsong Ye, Guangxu He, Fangfang Song, Chanyuan Jin

**Affiliations:** 1 Section of Human Anatomy, Department of Neuroscience, University of Padova, Padua, Italy; 2 Department of Women's and Children's Health, University of Padova, Padua, Italy; 3 Department of Surgery, Oncology and Gastroenterology, University of Padova, Padua, Italy; 4 Center of Regenerative Medicine, Department of Stomatology, Renmin Hospital of Wuhan University, Wuhan, China; 5 Sydney School of Dentistry, The University of Sydney, Sydney, NSW, Australia; 6 Trauma Center, Second Xiangya Hospital, Central South University, Changsha, China; 7 The Second Clinical Division of Peking University School and Hospital of Stomatology, Beijing, China; 8 State Key Laboratory of Oral and Maxillofacial Reconstruction and Regeneration, Key Laboratory of Oral Biomedicine Ministry of Education, Hubei Key Laboratory of Stomatology, School and Hospital of Stomatology, Wuhan University, Wuhan, China

**Keywords:** bone formation, bone regeneration, bone health, osteogenic differentiation, regenerative medicine, stem cells

Successful bone regeneration for large defects, particularly those involving substantial bone loss or occurring at load-bearing sites, remains a significant clinical challenge (1, 2). Bone remodeling and turnover result from coupled bone resorption/formation (3); however, there are events/conditions impairing this process. Additionally, with advancing age, bone structure undergoes mass decrease, microarchitecture modification, and a reduction/alteration in Mesenchymal Stromal Cells (MSCs) presence/phenotype (4, 5). Accelerated biological aging has been identified as an independent predictor of osteoporosis and premature mortality, highlighting the profound impact of systemic aging on skeletal health (6). Certainly, such conditions require additional support to re-establish bone architecture and function (7) and in this critical context tissue engineering (1) and stem cell-based therapies have emerged as appealing for bone regeneration. In particular, dental pulp stem cells (DPSCs) have garnered significant attention due to their easy accessibility, minimal collection-associated risks, and demonstrated potential for osteogenic differentiation, as well as their secretion of developmental-stage–specific bioactive components, including extracellular vesicles (EVs). In addition, MSCs cell lysates have also been explored for their therapeutic potential Liu et al.. Furthermore, the application of dental pulp stem cell-conditioned medium (DPSC-CM) has shown superior efficacy in preserving autologous bone grafts and enhancing bone healing *in vivo*, suggesting its potential as a novel osteogenic and angiogenic therapeutic strategy (11).

Within tissue engineering strategies, several materials can be adopted for fabrication of bone-like scaffolds with hierarchical microarchitecture. Further, MSCs play a leading role in this by promoting osteogenesis and modulating the immune response; as it occurs in the physiological environment (4). Finally, growth factors can be incorporated to guide and boost bone regeneration (8). Overall, the primary aim of tissue engineering is to overcome conventional therapeutic strategies limitations; however, to achieve this, a clear understanding of the mechanisms/events regulating bone regeneration, the behavior of bone-forming cells, and their activity within inflammatory environments still needs to be elucidated (12), thus representing the goal of this special issue (Figure 1).

**Figure 1:**
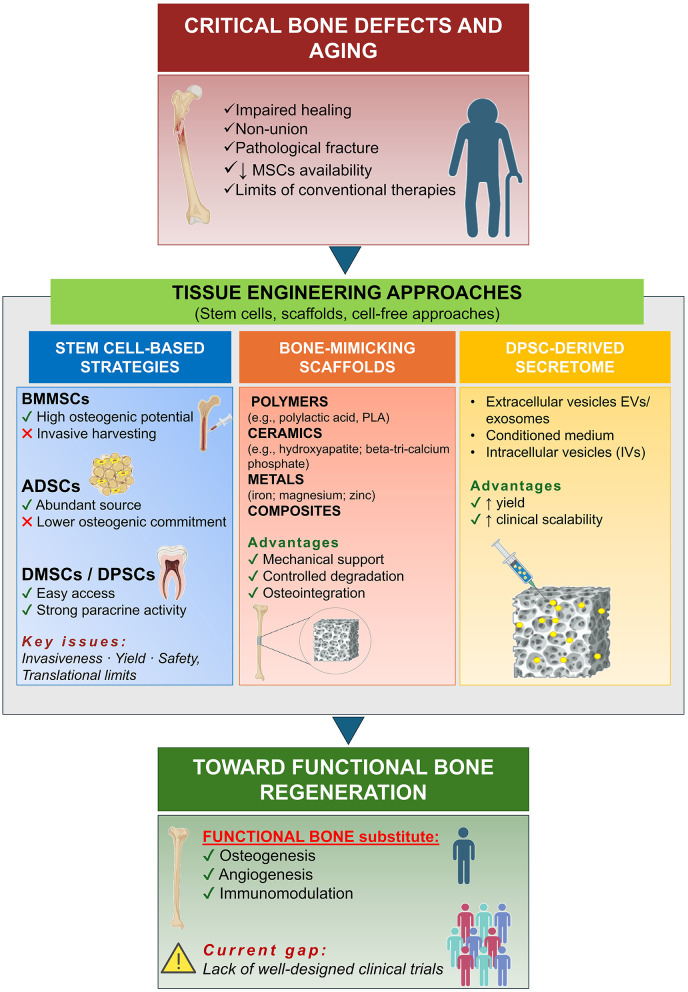
Tissue engineering for functional bone regeneration: schematic overview. Critical bone defects, particularly in aging patients, are characterized by impaired remodeling and reduced mesenchymal stem cell (MSCs) availability with a negative impact over conventional therapies. Different stem cell sources are considered for bone recovery: bone marrow (BMMSCs), adipose tissue (ADSCs), and dental tissue/dental pulp stem cells (DMSCs/DPSCs), are the most studied, each with specific advantages and translational limitations. Bone-mimicking scaffolds composed of polymers, ceramics, metals, or composites provide mechanical support, controlled degradation, and osteointegration. Cell-free approaches based on DPSC-derived secretome [e.g., extracellular vesicles (EVs), exosomes, conditioned medium, and intracellular vesicles (IVs)] are associated with enhanced yield and improved clinical scalability. Overall, tissue engineering approaches aim to promote osteogenesis, angiogenesis, and immunomodulation toward functional bone regeneration, although well-designed clinical trials are still needed to support clinical translation (Created with BioRender.com).

In their Review article, Jiao et al. provide a comprehensive overview of bone tissue engineering approaches. Preliminarily, various stem cell sources have been explored for bone regeneration, highlighting the specific advantages and limitations. MSCs, including bone marrow (BMMSCs), adipose tissue (ADSCs), and dental tissue stem cells (DMSCs), are the most studied. BMMSCs are easily induced toward osteogenesis but collection is painful with low yield; ADSCs are abundant and minimally invasive to isolate but have lower osteogenic potential and tend to differentiate into adipocytes; DMSCs are easily harvested with high proliferation but lower osteogenic capacity. Embryonic stem cells are pluripotent with unlimited self-renewal, useful for disease modeling, but their use is limited by ethical issues, low availability, and risks of mutation or teratoma formation. Induced pluripotent stem cells overcome ethical issues, are versatile, and are compatible with human leukocyte antigens; nevertheless, they carry risks of genomic instability and tumorigenesis, along with high associated costs. Cell-free therapies using MSC-derived EVs also promote bone regeneration with lower immune risk, but production and donor variability remain a challenge. Toward this broad overview of cells and their derivatives for bone regeneration, attention was also given to biomaterials. Bone scaffolds are made of polymers (e.g., polylactic acid, PLA), ceramics (e.g., hydroxyapatite; beta-tri-calcium phosphate), metals (iron; magnesium; zinc), and composites. In particular, composites combine ceramics bioactivity with the polymers' flexibility, optimizing scaffold strength, degradation, osteointegration and support for bone tissue growth. An interesting overview of human clinical trials employing MSCs embedded in scaffolds for bone regeneration is reported. The 14 studies considered (138 patients), involved BMMSCs, DMSCs, and ADSCs combined with natural, synthetic, and hybrid scaffolds. Results showed significant bone regeneration, improved density, soft tissue healing, and implant integration, with low, mostly mild adverse events.

Chen et al. performed bibliometric analysis of research on MSC-based bone regeneration being conducted globally (years: 2013–2023). A total of 8,070 articles from the Web of Science Core Collection were considered and systematically evaluated. Publication output showed an increasing trend overall, peaking in 2020. China and the United States were the leading contributors in terms of publication volume and collaboration networks. The dominant research fields were cell biology, material science and engineering. Keyword and citation analyses highlighted emerging hotspots including EVs and bone tissue engineering. Interestingly, it appears that the research focus has progressively shifted from basic MSC biology to translational and clinical applications. Overall, this study provides a comprehensive overview of current trends and future directions in MSC-based bone regeneration research.

Liu et al., describe the major signaling pathways involved in bone tissue regeneration (Hedgehog, Notch, WNT, and BMP/TGF-β pathways and others). The review identifies DPSCs as a promising therapeutic option for bone regeneration mediated by osteogenic differentiation, paracrine mechanisms and secretion of EVs. Interestingly, cell-free therapeutic strategies, based on the DPSC secretome, are gaining importance as they can overcome stem cell transplantation safety issues. Furthermore, combining DPSC-derived secretome with biomaterials seems to enhance regenerative outcomes. These approaches are promising despite being mostly at a preclinical stage.

Finally, Zhao et al. described trends in MSCs-based therapies for intervertebral disc degeneration treatment, through a bibliometric analysis. A steady increase in research activity was demonstrated in this area from the 931 publications evaluated (years 2000–2024). China and the United States confirmed their leading role in overall publication output (as in Chen et al.), whereas the United Kingdom showed the highest citation impact. Additionally, early studies mainly focused on gene expression and extracellular matrix regulation, and then interest moved to autophagy, oxidative stress, apoptosis, and cell-free strategies involving EVs and exosomes. Significantly, this evolution aligns with the recent emergence of novel nanovesicle platforms. Beyond conventional EVs, intracellular vesicles (IVs) derived from cell lysates represent a promising advancement. IVs not only exhibit bioequivalence to traditional EVs in promoting tissue regeneration and modulating inflammation but also offer a substantially higher (16-fold) acquisition efficiency, addressing a critical bottleneck for clinical translation (9). IVs are increasingly recognized as novel nanovesicles with superior potential for translational medicine and clinical applications compared to standard EVs (10). Overall, future research is expected to improve knowledge on MSCs for development of cell-free therapies, also targeting molecular pathways driving disc degeneration and regeneration (Zhao et al.).

To date, effective bone tissue regeneration suffers from limits of conventional therapies. Considering this critical scenario, tissue engineering approaches have emerged, providing promising solutions. More recently, cell-free therapies (EVs, stem cells conditioned media and secretome, or scaffolds conditioned with secretome), have gained increasing attention as they avoid the complexities and risks associated with live cell transplantation. Despite these advances, a critical gap remains in translating these strategies into clinical practice. Well-designed, large-scale clinical trials are still lacking, highlighting the urgent need for rigorous studies to prove their safety and efficacy in humans.

## References

[1] Li JJ Ebied M Xu J Zreiqat H. Current approaches to bone tissue engineering: the interface between biology and engineering. Adv Healthc Mater. (2018) 7:e1701061. doi: 10.1002/adhm.201701061 29280321

[2] Sun J Chen C Zhang B Yao C Zhang Y. Advances in 3D-printed scaffold technologies for bone defect repair: materials, biomechanics, and clinical prospects. Biomed Eng Online. (2025) 24:51. doi: 10.1186/s12938-025-01381-w 40301861 PMC12042599

[3] Unnanuntana A Rebolledo BJ Khair MM DiCarlo EF Lane JM. Diseases affecting bone quality: beyond osteoporosis. Clin Orthop Relat Res. (2011) 469:2194–206. doi: 10.1007/s11999-010-1694-9 21107923 PMC3126973

[4] Mi B Xiong Y Knoedler S Alfertshofer M Panayi AC Wang H . Ageing-related bone and immunity changes: insights into the complex interplay between the skeleton and the immune system. Bone Res. (2024) 12:42. doi: 10.1038/s41413-024-00346-4 39103328 PMC11300832

[5] Stocco E Barbon S Piccione M Belluzzi E Petrelli L Pozzuoli A . Infrapatellar Fat pad stem cells responsiveness to microenvironment in osteoarthritis: from morphology to function. Front Cell Dev Biol. (2019) 7:323. doi: 10.3389/fcell.2019.00323 31921840 PMC6914674

[6] Zhang R Zhong W Gao Y Duan X Ye Q. Biological age acceleration predicts osteoporosis and reduced longevity in a large prospective cohort. Bone. (2025) 200:117609. doi: 10.1016/j.bone.2025.117609 40816510

[7] Dec P Modrzejewski A Pawlik A. Existing and novel biomaterials for bone tissue engineering. Int J Mol Sci. (2022) 24:529. doi: 10.3390/ijms24010529 36613972 PMC9820083

[8] Jagadale S Damle M Joshi MG. Bone tissue engineering: from biomaterials to clinical trials. Adv Exp Med Biol. (2025) 1479:73–115. doi: 10.1007/5584_2024_841 39881051

[9] Duan X Zhang R Feng H Zhou H Luo Y Xiong W . A new subtype of artificial cell-derived vesicles from dental pulp stem cells with the bioequivalence and higher acquisition efficiency compared to extracellular vesicles. J Extracell Vesicles. (2024) 13:e12473. doi: 10.1002/jev2.12473 38965648 PMC11223992

[10] Ye Q Zhang R. Intracellular vesicles: novel nanovesicles superior to extracellular vesicles in translational medicine and clinical applications. Nano TransMed. (2024) 3:100044. doi: 10.1016/j.ntm.2024.100044

[11] Liu Y Liu Y Ye Z Wang X Li J Mei PL . Application of dental pulp stem cell-conditioned medium combined with deep cryopreservation of autologous cranial flaps. Stem Cell Res Ther. (2025) 16:272. doi: 10.1186/s13287-025-04407-1 40457321 PMC12131630

[12] Pajarinen J Lin T Gibon E Kohno Y Maruyama M Nathan K . Mesenchymal stem cell-macrophage crosstalk and bone healing. Biomaterials. (2019) 196:809. doi: 10.1016/j.biomaterials.2017.12.025 29329642 PMC6028312

